# Amyloid-β Oligomer Specificity Mediated by the IgM Isotype – Implications for a Specific Protective Mechanism Exerted by Endogenous Auto-Antibodies

**DOI:** 10.1371/journal.pone.0013928

**Published:** 2010-11-10

**Authors:** Malin Lindhagen-Persson, Kristoffer Brännström, Monika Vestling, Michael Steinitz, Anders Olofsson

**Affiliations:** 1 Department of Medical Biochemistry and Biophysics, Umeå University, Umeå, Sweden; 2 Department of Pathology, Hebrew University Hadassah Medical School, Jerusalem, Israel; Mental Health Research Institute of Victoria, Australia

## Abstract

**Background:**

Alzheimers disease (AD) has been strongly linked to an anomalous self-assembly of the amyloid-β peptide (Aβ). The correlation between clinical symptoms of AD and Aβ depositions is, however, weak. Instead small and soluble Aβ oligomers are suggested to exert the major pathological effects. In strong support of this notion, immunological targeting of Aβ oligomers in AD mice-models shows that memory impairments can be restored without affecting the total burden of Aβ deposits. Consequently a specific immunological targeting of Aβ oligomers is of high therapeutic interest.

**Methodology/Principal Findings:**

Previously the generation of conformational-dependent oligomer specific anti-Aβ antibodies has been described. However, to avoid the difficult task of identifying a molecular architecture only present on oligomers, we have focused on a more general approach based on the hypothesis that all oligomers expose multiple identical epitopes and therefore would have an increased binding to a multivalent receptor. Using the polyvalent IgM immunoglobulin we have developed a monoclonal anti-Aβ antibody (OMAB). OMAB only demonstrates a weak interaction with Aβ monomers and dimers having fast on and off-rate kinetics. However, as an effect of avidity, its interaction with Aβ-oligomers results in a strong complex with an exceptionally slow off-rate. Through this mechanism a selectivity towards Aβ oligomers is acquired and OMAB fully inhibits the cytotoxic effect exerted by Aβ(1-42) at highly substoichiometric ratios. Anti-Aβ auto-antibodies of IgM isotype are frequently present in the sera of humans. Through a screen of endogenous anti-Aβ IgM auto-antibodies from a group of healthy individuals we show that all displays a preference for oligomeric Aβ.

**Conclusions/Significance:**

Taken together we provide a simple and general mechanism for targeting of oligomers without the requirement of conformational-dependent epitopes. In addition, our results suggest that IgM anti-Aβ auto-antibodies may exert a more specific protective mechanism in vivo than previously anticipated.

## Introduction

Amyloid is today linked to more than twenty-five different syndromes, of which the neurodegenerative disorder Alzheimer's disease (AD) represents the most prominent example. Amyloid depositions in AD patients are largely composed of the Aβ-peptide, derived from the proteolytic cleavage of the amyloid precursor protein (APP). Aβ peptides of 39–43 residues all have clinical relevance, but Aβ(1–40) and Aβ(1–42) represent the most abundant variants. Aβ(1–42), in particular, has a significantly higher propensity to aggregate, and has been strongly implicated in the etiology of AD [1].

Although the histopathological features of Aβ depositions (plaques) are striking, the correlation between Aβ depositions and the clinical symptoms of AD is weak [2]. Small and soluble Aβ assemblies, known as Aβ oligomers, correlate better with negative clinical status and have been suggested to be the Aβ species primarily responsible for cytotoxicity. The structural features of Aβ oligomers are largely unknown. Aβ-targeting immunization, both passive and active, has been studied in AD mouse models and both types of immunizations have shown a dramatic improvement regarding memory and behavior [3], [4], [5], [6], [7], [8]. Interestingly, passive Aβ vaccination improved cognitive performance within days, without reducing plaque burden, suggesting that the removal of soluble Aβ is an adequate treatment to restore cognitive function [6].

Aβ and its precursor APP has both been suggested to have important roles concerning neuroprotection and neurotrophicity [9], [10], [11]. Therefore, to avoid interfering with the homeostatic functions of amyloid proteins, a therapeutic approach focused on clearance of Aβ should preferentially target Aβ oligomers while preserving Aβ monomers and APP. Intriguingly, conformational-dependent anti-Aβ antibodies, of the IgG isotype, have been shown to selectively target Aβ oligomers, suggesting that an oligomer-specific architecture exists [12], [13], [14], [15]. However, to avoid the difficult task of identifying a specific architecture, only present within the oligomeric form, we have employed a different rationale based on the assumption that all oligomeric variants of Aβ must expose several similar epitopes. The binding of a multivalent target to a corresponding multivalent receptor will be influenced by the avidity that, as opposed to affinity, represents the product of all single valence interactions. Avidity and affinity are, however, correlated, as the strength of avidity depends both on the affinity of each interaction, as well as on the number of interactions. We propose that an IgM antibody, containing 10 identical binding sites, fulfils the requirements to be a multivalent receptor for oligomers. We have therefore generated an oligomer-specific IgM anti-Aβ antibody (OMAB) that binds Aβ oligomers with a high specificity and a high affinity, whereas its binding to Aβ monomers and dimers is comparatively weak. OMAB has a high specificity for oligomeric Aβ even in a complex medium (containing many different forms of Aβ), demonstrated by its ability to completely block, at a highly substoichiometric ratio, the cytotoxic effects exerted by oligomeric Aβ(1–42). The specificity of a previously identified human anti-Aβ IgM antibody (L11.3) was also evaluated in the current study, and the results were analogous to those obtained with OMAB, demonstrating a high affinity for Aβ oligomers as compared to Aβ monomers and dimers.

Interestingly, anti-Aβ auto-antibodies of both IgG and IgM isotypes are commonly found in the sera of the healthy human population [16], [18], [19], [20], [21], [22], [23], [24]. To further study the Aβ binding pattern of endogenous anti-Aβ antibodies having the IgM isotype, we have screened a set of plasma samples from healthy donors. These studies found that the isolated endogenous anti-Aβ IgM antibodies all displayed a much higher affinity towards the oligomeric Aβ as compared to monomeric Aβ.

Our findings suggest a simple mechanism whereby avid IgM binding can be used to specifically target oligomeric assemblies of Aβ. With such an approach, antibody binding is not restricted to specific epitope conformations: the epitope recognized by the IgM antibody need only be exposed within the oligomeric state. Moreover, our findings suggest a more specific and possibly protective role for the abundant spectra of anti-Aβ IgM auto-antibodies present in humans.

## Materials and Methods

### Ethical statement

The use of all human samples, within this study, was verbally approved by the donors. The study has been fully anonymized and no data regarding specific individuals has been recorded. This implies no possibility to identify and connect specific individuals to the acquired result. All work and procedures involving human samples was approved by the Committee of Research involving Human subjects of the Hebrow University Hadassah medical school, Jerusalem, Israel, according to the declaration of Helsinki (permit number: 211-09.02.07). All immunisations of mice was approved by the Umeå Ethical Board of Animal Research, Västerbottens län, hovrätten övre norrland, box 384, 901 08, Umeå, Sweden, according to the declaration of Helsinki (permit number A47-07).

### Immunization and cell fusion

Mice (BALB/c, female, 8–10 weeks old) were immunized with the recombinant Aβ(1–40) (Agrisera AB, Vännäs, Sweden). Six weeks following immunization, sera were tested for antibodies against Aβ. Mice testing positive for anti-Aβ antibodies were then given booster i.v. injections of 50 µg of Aβ(1–40). Fusion of spleenocytes with mouse B-cell myeloma cells was performed 3 days later according to standard procedures and cells were seeded in 96-well plates at a density of 300 000 cells/well. Selection for hybrid cells was performed using Dulbecco's Modified Eagle Medium/DMEM) with high glucose (4500 mg/L D-Glucose) containing 10% (v/v) fetal calf serum and hypoxanthine/aminopterin/thymidine (Gibco). Supernatants from wells with growing cells were screened for antibodies reactive to Aβ. Isotype determination was performed using the IsoQuick™ strips for Mouse Monoclonal Isotyping (Sigma-Aldrich, Germany).

### Cell culture

SH-SY5Y neuroblastoma cells were obtained from the European Collection of Cell Cultures (Center for Applied Microbiology and Research, Salisbury, Wiltshire, UK). Cells were cultured in MEM with Earle's salts (Gibco), supplemented with 10% (v/v) foetal bovine serum, 2 mM l-glutamine (Gibco), 100 units/ml penicillin, 100 µg/ml streptomycin (Gibco), and 1% non-essential amino acid solution (Gibco). Cultures were maintained in an incubator at 37°C, with a humidified atmosphere of 5% (v/v) CO2/air.

### Cytotoxicity measurement

Aβ(1–42) (AlexoTech AB, Umeå, Sweden) was dissolved in 10 mM NaOH to 160 µM and further diluted with 4X MEM and H2O to 40 µM, pH 7.4. The Aβ(1–42) sample was further diluted with 1X MEM to the desired concentration and supplemented with 2 mM l-glutamine, 100 units/ml penicillin, 100 µg/ml streptomycin (Gibco), 1% MEM non-essential amino acid solution (Gibco), 1% MEM vitamins solution (Gibco), and 1% MEM amino acids solution (Gibco). Aβ(1–42) was allowed to incubate with SH-SY5Y cells in the absence or presence of OMAB. Cell viability was measured after 48 hours using a resazurin reduction test, a test where blue non-fluorescent resazurin is reduced to pink highly fluorescent resorufin by living cells [25]. Fluorescence, directly proportional to cell viability, was measured in each well with a Tecan Infinite plate reader set to 535 nm excitation and 595 nm emission. Cytotoxicity is presented as a percentage of the fluorescence of control cells incubated with MEM only. Statistical analysis was performed using GraphPad Prism version 5.01 for windows (GraphPad Software, San Diego, California, USA). Viability of cells incubated with Aβ oligomers in the presence or absence of OMAB was analyzed using One-Way ANOVA with Dunnett's post test. Statistical significance was set to a p-value ≤0.001.

### Oligomer preparation and size-exclusion chromatography (SEC)

Due to the labile nature of Aβ oligomers their preparation must be carefully monitored. Dissolution of the peptide is perhaps the most critical step in the procedure where it is of paramount importance to achieve an effective monomerisation. Presence of fibrillar seeds otherwise results in an accelerated conversion into amyloid. There are several methods described in the literature, where the peptide is dissolved using organic solvents followed by dilution. However, in our hands these methods frequently result in impurities where fibrillar seeds prevent population of oligomers. We have therefore focused on a commonly used alternative where the peptide is dissolved at high pH followed by adjustment of the pH using the buffer of choice. Using the cytotoxic potential as readout, we have optimized the procedure for oligomer preparation under cell-culture conditions with respect to Aβ concentration and time of incubation. Aβ(1–42) (AlexoTech AB, Umeå, Sweden) was dissolved in 10 mM NaOH to a concentration of 160 µM. The Aβ(1–42) sample was further diluted to 10 µM or 50 µM (the higher concentration was used regarding subsequent screen for human auto-antibodies) in PBS and the pH was adjusted to 7.4. Following incubation for 1 hour at RT (OMAB) or 2 hours at 4°C (auto-antibodies), the samples were separated by size-exclusion chromatography (SEC) (GE Superdex-G75 10/30) at 4°C. To determine the molecular weight of the Aβ species, a gel filtration protein standard (Bio-Rad) was separated by SEC under the same conditions as the Aβ samples.

### Indirect ELISA using OMAB

The OMAB antibody, diluted to 10 µM/ml in PBS, was adsorbed to Nunc-Immuno MaxiSorp plates (Nunc, Roskilde, Denmark). Aβ-fractions collected from the SEC were allowed to bind to OMAB plates for 20 minutes at 0°C. Bound Aβ was detected using a polyclonal rabbit anti-Aβ antibody (AS08 328, Agrisera AB, Vännäs, Sweden) at a 1∶1000 dilution followed by an anti-rabbit HRP-conjugated secondary antibody at a 1∶5000 dilution (GE healthcare). EC-Blue (Medicago, Uppsala, Sweden) was used as a substrate for HRP and the signal was detected by measuring the absorbance at 450 nm. The signal of EC-Blue can be compared within, but not between plates. Blocking solution and antibody-dilutions were made with 5% Non-fat dry milk in PBST and all washes were performed with PBS containing 0.1% Tween-20 (PBST).

### Indirect ELISA using anti-human IgM

The anti-human IgM antibody (Thermo scientific, Rockford, USA), diluted to 10 µg/ml in PBS, was adsorbed to Nunc-Immuno MaxiSorp plates (Nunc, Roskilde, Denmark). Human plasma from eight anonymous healthy donors was diluted 4X in PBS, added to the wells, and incubated for 1 hour at RT. During this incubation step, IgM auto-antibodies present in the plasma were allowed to bind to the anti-IgM antibodies previously adsorbed to the plate. Aβ-fractions collected from the SEC were thereafter allowed to bind to the IgM auto-antibodies for 20 minutes at 0°C. Detection, blocking, and washes were performed as described previously.

### Epitope mapping and determination of affinity using surface plasmon resonance (SPR)

The OMAB antibody was immobilized at a density of 10 000–15 000 RU to a CM5 chip (GE Healthcare) using standard amine-coupling chemistry at pH 6. Analysis of Aβ binding was performed at a flow rate of 50 µl/min in PBS at 25°C. Sensograms were corrected for non-specific interactions to a reference surface, and by double referencing [26]. For the determination of affinity constants, Aβ(1–16) was injected at concentrations ranging from 125 nM to 6 µM. The dissociation constant for Aβ(1–16) was determined by fitting the response at the end of each of the association phases to a single-site binding isotherm. Epitope mapping of OMAB was performed at a peptide concentration of 5 µM.

## Results

### Characterization of OMAB

**Figure 1:**
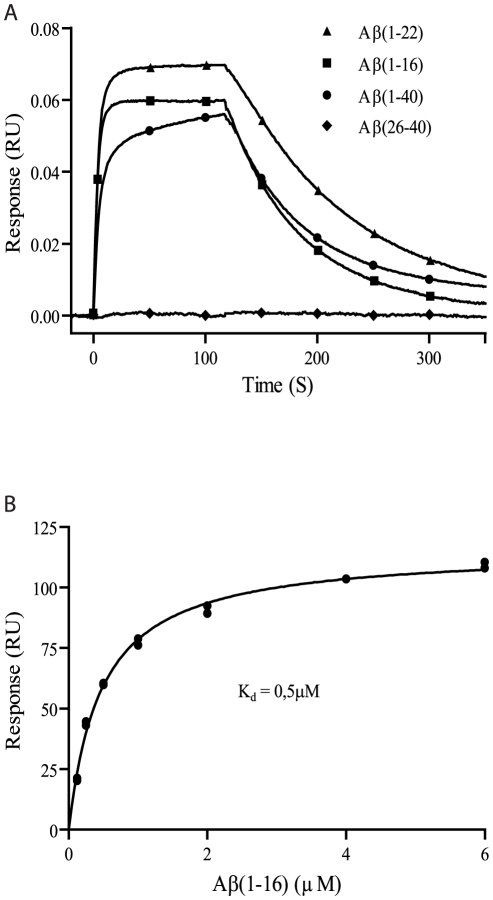
OMAB binds to the N-terminus of Aβ with an affinity of 0.5 µM. OMAB was immobilized to a CM5 chip and tested for biding to (A) Aβ peptides of different lengths at a concentration of 5 µM. The responses were normalized by the molecular weight of each peptide. (B) Aβ(1–16) ranging in concentration from 125 nM to 6 µM. All experiments were performed in PBS at 25 &degC. The dissociation constant for Aβ(1–16) was determined by fitting the response at the end of each of the association phases to a single-site binding isotherm.

Monoclonal antibodies obtained from hybridomas were screened for reactivity against different forms of Aβ using dot blots and indirect ELISA (data not shown). A particular clone, denoted OMAB, fulfilled the requirements of both being of the IgM isotype and exhibit Aβ binding and was therefore characterized further. Using SPR, a technique that facilitates detailed monitoring of binding properties, the affinities between different monomeric Aβ fragments and the OMAB antibody were studied. An interaction was detected between OMAB and Aβ(1–16), Aβ(1–22), and Aβ(1–40), while no reactivity was seen between OMAB and Aβ(26–40) (Fig. 1A). Similar results were obtained using ELISA (data not shown). The dissociation rates were essentially the same for all interactions (except for OMAB with Aβ(26–40)), with a T_1/2_ corresponding to about 75 s, which implies a similar, monovalent binding of OMAB to the different Aβ fragments. The reactivity pattern of OMAB suggests that the specific target epitope of OMAB is located within Aβ residues 1–16, a flexible portion of the Aβ sequence that becomes partly structured within mature fibrils [27], [28]. The dissociation constant between Aβ and OMAB was investigated using monomeric Aβ(1–16), an Aβ fragment not prone to aggregate. The dissociation constant was determined to 0.5 µM by fitting to a single site binding isotherm (Fig. 1B).

### Affinity of OMAB for different Aβ assemblies

**Figure 2:**
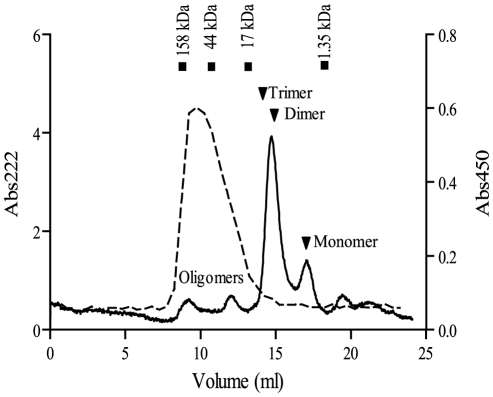
OMAB selectively reacts with oligomeric Aβ. A sample containing different oligomeric species of Aβ(1–42) was separated by SEC (-). Fractions, collected throughout the SEC, were tested in an indirect ELISA for reactivity with OMAB (---). In connection to the SEC of Aβ(1–42), a sample containing proteins with known molecular weights was analyzed. The exclusion volume of the peak maxima of these proteins is illustrated in the figure (▪).

Aβ(1–42) peptides rapidly self-assemble into a range of different small and soluble oligomers at 37°C in PBS, a reaction easily monitored using SEC. The predominant species are monomers, dimers, and trimers; higher multimers are also detected, but they are present at considerably lower concentrations. Using an indirect ELISA setup where OMAB was probed against various Aβ species, we showed that OMAB exhibited considerable selectivity towards oligomeric species. OMAB identified oligomeric Aβ species with a molecular weight between 15–100 kDa, but failed to detect the much more abundant fractions of monomers and dimers (Fig. 2). The second anti-Aβ antibody, AS08 328, used in the indirect ELISA, binds Aβ within the N-terminus, as does OMAB. The N-terminal localization of these antibodies is a deliberate choice since the N-terminal part of Aβ that remains exposed after aggregation. It is important to note that OMAB and AS08 328 competed for Aβ to only a minor extent (20%, data not shown).

**Figure 3:**
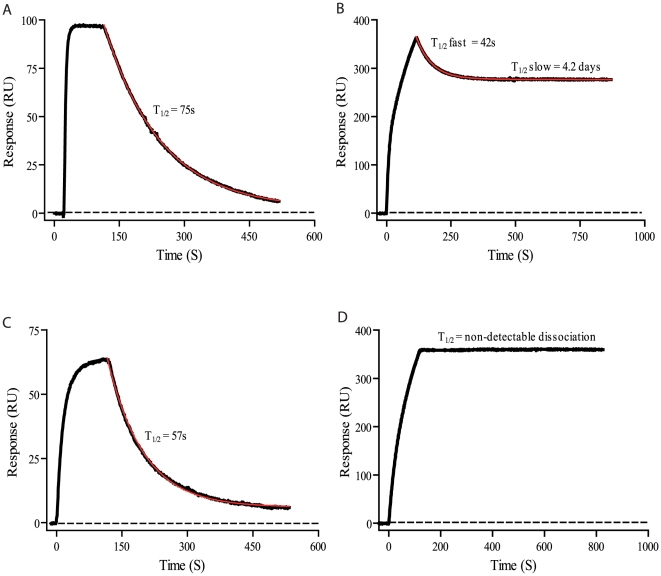
A complex between Aβ oligomers and OMAB display very slow dissociation rate. Different samples of Aβ(1–40) and Aβ(1–42) was separated by SEC. (A) The Aβ(1–40) monomeric fraction or (B) Aβ(1–40) the oligomeric or (C) monomeric fraction of Aβ(1–42) or (D) Aβ(1–42) the oligomeric fraction were analyzed for binding to OMAB, immobilized to a CM5 chip. The dissociation of (A) monomeric Aβ(1–40) was fitted to a single exponential decay (red line) with a rate constant of 0.0092 s and a T1/2 of 75 s, and of (B)(1–40) oligomeric Aβ to a double exponential decay (red line) with rate constants of 0.016 and 1.88 * 10-6, and with a T1/2 of 42 s and 4.2 days, respectively. (C) monomeric Aβ(1–42) was fitted to a single exponential decay (red line) with a rate constant of 0.012 s and a T1/2 of 57 s for the oligomeric fraction of Aβ1-42 (D) no dissociation was detected due to a to very strong interaction. All experiments were performed in PBS at 25°C.

Indirect ELISA illustrated the binding pattern of OMAB to Aβ, but failed to elucidate the binding kinetics. Using SPR, the binding kinetics between OMAB and the different Aβ species was examined. OMAB immobilized on the surface of a biocore CM5 chip, was probed with different Aβ species obtained through SEC. A dramatic difference in dissociation rates was observed between the different species. The monomer-dimer fractions displayed, in accordance with the different monomeric Aβ variants, a very rapid dissociation rate that could be fitted to an exponential decay function with a T_1/2_ corresponding to 75 s, indicating a low binding affinity (Fig. 3a). A considerably stronger binding was observed to occur between OMAB and the oligomeric fractions containing Aβ(1–40) assemblies of approximately 10–20 Aβ peptides. The dissociation rate is clearly bi-modal and a double exponential decay model was needed for an accurate fit: the initial rapid phase has a T_1/2_ of 42s, likely reflecting a partial monovalent binding, while the second phase has a 5000X slower dissociation rate, with a T_1/2_ of 4.2 days (Fig. 3B). These results clearly illustrate a binding mechanism for OMAB strongly dependent upon avidity, where a low affinity at each antigen-binding site (i.e. between a single Aβ monomer and the antibody) is compensated for by an increase in the number of interactions. Regarding the Aβ(1–42) this effect was even more striking where no biphasic dissociation pattern was noted. The T_1/2_ of Aβ(1–42) monomers was measured to 57s (Fig. 3C), which is in the same range as monomeric Aβ(1–40). However, the binding between OMAB and Aβ(1–42) oligomers, constituted by approximately 10–20 Aβ peptides, was extremely strong and no dissociation could be noted throughout the duration of the experiment (Fig. 3D).

The lack of avidity to dimers, and to some extent, trimers may be explained by the relatively smaller size of these moieties, as the antigenic aggregate must be large enough to bridge the antigen binding sites on the IgM molecule.

### OMAB binds selectively to Aβ fibril ends and inhibits fibril growth

To further evaluate the binding specificity of OMAB, we probed its interaction towards mature Aβ fibrils. A convenient method for the monitoring of fibril polymerization in real time is to immobilize mature Aβ(1–40) fibrils on a Biacore CM5 sensor chip and incubate with an excess of monomeric Aβ(1–40) peptide, an event that will give a continuous propagation of the sensor chip signal as a result of fibril growth [29]. Upon the addition of OMAB to the immobilized Aβ fibrils, a strong, but highly substoichiometric binding was detected, which corresponded to a molar ratio of about 1∶2000 OMAB:Aβ. This substoichiometric binding suggested that only a small fraction of the Aβ fibrils expose the epitopes required for OMAB to bind, thus OMAB likely binds predominantly to exposed fibrillar ends rather than to the lateral fibril. This hypothesis is supported by the finding that immobilized fibrils, saturated with OMAB, were unable to bind monomeric Aβ (data not shown). Thus, OMAB effectively inhibited the Aβ polymerization process.

### OMAB effectively inhibits the cytotoxicity of Aβ in a cell-viability assay

**Figure 4:**
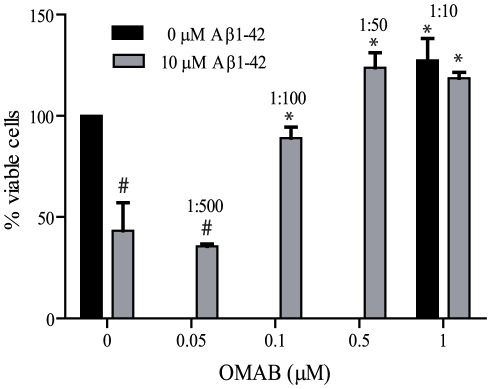
OMAB inhibits the cytotoxicity of Aβ(1–42) in a substoichiometric ratio. OMAB was incubated with SH-SY5Y cells in the presence or absence of Aβ(1–42). After 48 hours, cell viability was detected using resazurin. # = p<0.001 compared to control cells and * = p<0.001 compared to cells treated with only Aβ(1–42).

The cytotoxicity of Aβ was studied using a cell-based viability assay. Freshly prepared Aβ(1–42) was added to cultured SH-SY5Y cells, resulting in 35% of cells remaining viable after 48 hours of Aβ exposure. Co-incubation of Aβ and OMAB essentially eliminated the cytotoxic effect at a substoichiometric OMAB:Aβ ratio corresponding to 1∶50 (Fig. 4).

### Human IgM auto-antibodies can selectively target the oligomeric fraction of Aβ

**Figure 5:**
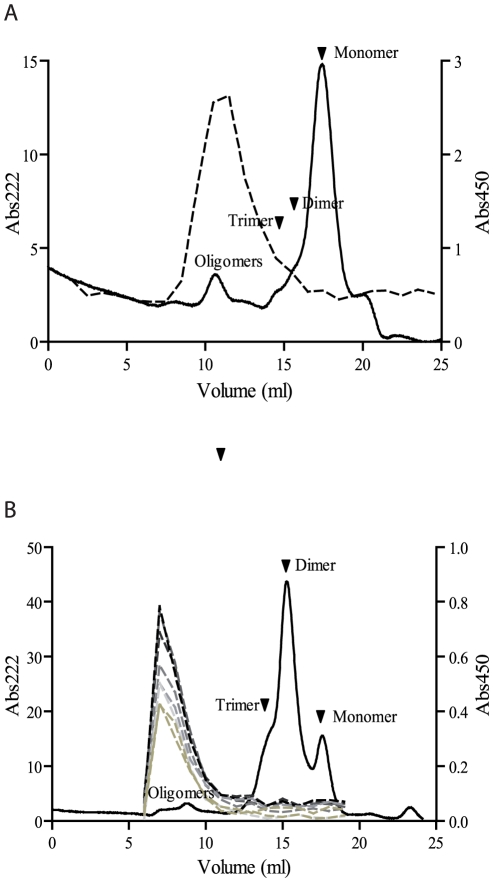
Human IgM auto-antibodies specifically detect Aβ oligomers. A sample containing different oligomeric species of Aβ(1–42) was separated by SEC (-). Fractions, collected throughout the SEC, were tested in an indirect ELISA for reactivity with (A) L11.3 (---) or (B) plasma from eight different human donors (---). Before adding L11.3 or plasma, the ELISA plate was coated with an anti-human IgM antibody to ensure that only IgM auto-antibodies were allowed to react with the Aβ species. The level of endogenous anti-Aβ antibodies is low in the plasma and to ensure a measurable response 5X more total Aβ was applied on the SEC column which explains the different elution profiles.

Auto-antibodies against Aβ are common in humans, and both IgG and IgM isotypes have been described [16], [18], [19], [20], [21], [22], [23], [24]. In a previous study, an immortalized cell line that produces an anti-Aβ human IgM monoclonal antibody was established from a healthy human blood donor [17]. This specific antibody, denoted L11.3, was able to prevent the phenotype of impaired spatial cognition in aged SAMP mice, similar to previous findings using IgG antibodies [6], [7], [30], [31]. To compare the binding properties of the L11.3 antibody with the mouse-derived OMAB, we examined L11.3 in our indirect ELISA system. Like OMAB, L11.3 showed a strong selectivity for oligomeric Aβ (Fig. 5A).

The reaction pattern of human IgM against Aβ was further studied by investigating plasma from a group of healthy donors. Similar to both the murine OMAB and the human L11.3 monoclonal antibodies, human plasma IgM bound oligomeric Aβ, while Aβ monomers and dimers were omitted (Fig. 5B).

## Discussion

Self-assembly of monomeric Aβ peptides is a multifactorial process, where the resulting aggregates can differ in both morphology and molecular structure. This difference is reflected in the ability of the different Aβ aggregates to exert a pathological effect. Compelling evidence suggests that small, soluble oligomeric species, and not large, insoluble aggregates, are the most cytotoxic Aβ species. This hypothesis has been supported by cell-culture viability assays and through numerous animal experiments, where several independent investigations have described memory impairment in APP transgenic mice to precede the detection of Aβ depositions [32], [33], [34], [35]. Soluble Aβ oligomers, heterogeneous assemblies vastly ranging in size, were initially described as a soluble pool of Aβ that remained in solution after ultra-centrifugation [36]. This Aβ pool was subsequently divided into subfractions from 10 to 100 kDa using filters with different molecular weight cut-offs. To date, a variety of different soluble Aβ species have been characterized and their physiological effects characterized (for a recent review, see Shankar et al [37]). Thus, a large accumulation of evidence implicates soluble Aβ oligomers as highly promising therapeutic targets.

There are different approaches to the targeting of soluble Aβ oligomers, one approach being immunization. Promising results have been obtained with both active and passive immunization in AD mice [3], [4], [5], [6], [7], [8]. However, active vaccination performed in human AD patients has, to date, resulted in only moderate clinical improvements and, in a small number of patients, been associated with a pathological auto-immune T-cell response resulting in encephalitis [38]. In this respect passive vaccination may provide a safer alternative.

Administration of an anti-Aβ antibody can reverse spatial memory deficits in AD mice after only a few days of treatment without affecting the total burden of deposited Aβ [6], [39]. These findings suggest that neuronal death represents a late stage in the pathology of AD, and that earlier events in the disease may impair neuronal function without neurodegeneration. If a selective scavenger of Aβ oligomers could be administered at an early stage of the disease, AD-related brain damage could potentially be reduced.

In a recent publication, monomeric Aβ was shown to have a neuroprotective effect by preventing NMDA-induced exitotoxicity [10]. This finding is in accordance with previous results showing that Aβ levels increase following brain injury [40], [41], [42]. This finding implies that Aβ might have an important physiological role *in vivo*, a fact that should be considered when designing therapeutic strategies. It is important that the antibodies used in AD therapeutics have selective properties, and avoid binding both monomeric Aβ and native APP. Several different oligomer-specific IgG antibodies have been created to selectively target the specific fold that only Aβ oligomers are believed to possess [12], [13], [14], [15]. To avoid the difficulty of creating an antibody that recognizes a specific conformational architecture only present within the oligomers, we have chosen a different approach. Our technique is based on the rationale that all oligomers are multivalent, i.e. they potentially expose a number of identical epitopes. An efficient oligomer-specific receptor should, therefore be multivalent. However, to facilitate a dynamic screening for oligomers also in complex solutions in high excess of monomeric species, it is equally important to have a fast on- and off-rate at each single binding site. All species of Aβ exposing the target sequence will hence have the same ability to bind but, as a result of the avidity effect, the dissociation rate will be strongly reduced in oligomeric assemblies. Thus, the low affinity of each single interaction is compensated by a high avidity, the product of all single interactions. In other words the probability that all antigen-antibody interactions will dissociate simultaneously is exceedingly small therefore, if one interaction is dissociated, the others will remain associated, thus enhancing the probability that those dissociated will re-associate.

The multivalent IgM architecture fulfils the criteria of an effective oligomer receptor. In the current work, we characterized the oligomer specificity of OMAB, a newly designed monoclonal anti-Aβ antibody of the IgM isotype. Using indirect ELISA in combination with SEC-separated Aβ(1–42), we discovered OMAB to have a much higher affinity for Aβ oligomers than for Aβ monomers and dimers. SPR studies demonstrated that the avidity effect of OMAB induced a very strong binding to, and a high selectivity for, Aβ oligomers. The most striking effect was seen when comparing dissociation rates of the different Aβ species. The dissociation rate for Aβ monomers and dimers regarding Aβ(1–40) and Aβ(1–42) is rapid where T_1/2_ equals approximately 1 minute (Fig. 3A and 3C, respectively). On the contrary the corresponding oligomers, having an apparent size of a 10–20 mer, shows a dramatic decrease in dissociation rate where T_1/2_ for Aβ(1–40) equals 4.2 days, while T_1/2_ regarding Aβ(1–42) became to slow to be measured (Fig. 3B and 3D, respectively).

This finding suggests that the IgM isotype introduces the potential of multivalent binding, allowing oligomers to bind with a high affinity, while monomers and dimers bind only transiently with fast off-rate kinetics. When using IgM antibodies to trap Aβ oligomers, unwanted binding to Aβ monomers and APP is, to a great extent, omitted. It is important to emphasize that the high selectivity towards oligomers only can be obtained at a relevant time-scale, if the off-rate at each single binding site is high. A to high single valence affinity would slow down the process of identifying oligomers in a large excess of monomeric Aβ since the sites will be blocked by monomers. As a result the “scanning” effect for oligomeric species within the sample would be lost.

A potentially complication of generating oligomer-specific antibodies is the possible cross-reactivity to mature Aβ fibrils. SPR was used to investigate if OMAB detected amyloid fibrils. We found that only a small fraction of the OMAB antibody, corresponding to a molar ratio of 1∶2000 OMAB:Aβ, bound to the immobilized amyloid fibrils. We conclude that OMAB is unable to perform a lateral binding along the Aβ fibril, but instead binds to the more exposed fibrillar ends. This was verified through competition studies, where OMAB effectively inhibited a continuous polymerization of the fibril upon addition of free Aβ monomers.

Specific assemblies of Aβ(1–42) efficiently kill human neuronal cells in culture. Due to the kinetic instability of Aβ(1–42) in cell culture media, the initial oligomeric state of Aβ is not maintained throughout the duration of the experiment. As a consequence, it is difficult to elucidate the exact size of the cytotoxic Aβ assembly, as well as to determine the molar concentration of cytotoxic Aβ. We have shown that the addition of OMAB, in a substoichiometric ratio corresponding to 1∶50 (OMAB:Aβ monomer), completely blocks the neurotoxic effect exerted by the Aβ(1–42) peptide. This result also demonstrates the specificity of OMAB in a more complex matrix.

To date, a detailed characterization regarding the molecular properties and size of cytotoxic Aβ has been lacking. A cytotoxic effect of Aβ-derived diffusible ligands (ADDLs) has been described, comprising a rather broad spectrum of Aβ assemblies [43]. A cytotoxic effect has also been reported from spheroids, large Aβ assemblies with a molecular weight of 500 kDa [44]. An acute cytotoxic response is, however, a dramatic cellular effect and, *in vivo*, subtler changes may better reflect the pathological events underlying disease. Essentially all soluble forms of Aβ have the ability to inhibit long-term potentiation (LTP), a cellular correlate of memory (LTP) [13], [45], [46], [47], [48]. LTP is a long-lasting enhancement in signal transmission between two neurons and is one of several phenomena underlying synaptic plasticity, the ability of synapses to change their strength. As the ability to form memories is thought to be encoded by modifications in synaptic strength, LTP is considered to be a major cellular mechanism underlying learning and memory. In AD models, initial observations correlated impairment in spatial memory with the presence of Aβ nonamers and dodecamers [39], species that are readily detected by the OMAB antibody. However, impaired LTP, as well as a decreased dendritic spine density in the hippocampal dentate gyrus, has been noted prior to the detection of nonamers and dodecamers [49], suggesting that even smaller Aβ assemblies are able to exert a physiological effect *in vivo*. Engineered disulphide-linked dimers of Aβ have also been shown to inhibit LTP both *in vitro* and *in vivo* [47]. As OMAB fails to detect assemblies smaller than a trimer, possibly due to steric hindrance as a result of the difference in size, alternative smaller binding structures should be considered to accomplish an oligomer-specific effect targeting e.g. dimeric species.

The initial events regarding Aβ assembly may moreover be intracellular and design intracellular antibodies, delivered through an adenoviral vector, have resulted in partial clearance of Aβ(1–42) deposits in a mouse model for AD [50].

The IgM isotype has previously been evaluated *in vivo* using a transgenic AD mouse model [17]. Using brain injections of a human-derived anti-Aβ IgM antibody (L11.3), both memory and learning deficits could be restored. In this study, the injection of high intravenous doses of the IgM antibody resulted in a fraction crossing the blood brain barrier and ameliorating the learning deficiency [17]. In the current investigation, we have evaluated the binding properties of L11.3 using indirect ELISA and probed it against a spectrum of different Aβ assemblies in parallel with the murine-derived OMAB. Our results show L11.3 to exhibit a selectivity for the oligomeric Aβ fraction (Fig. 5A), similar to OMAB, and support the notion that the multiple antigen-binding sites of IgM induce a high specificity towards oligomeric Aβ assemblies. Epitope mapping of L11.3 demonstrated that antibody binding occurs within the 16 most N-terminal residues of Aβ (data not shown).

Endogenous autoantibodies against Aβ are common in humans [3], [4], [5], [6], [7], [8], [51], [52] and passive immunization using total isolate of human immunoglobulins, given to AD individuals has resulted in a slight cognitive improvement [53], [54]. Intriguingly, IgM anti-Aβ autoantibodies are found in essentially all individuals, although in different concentrations [17], [18], [23]. These human IgM autoantibodies have previously been studied with the aim of identifying differences between healthy controls and individuals affected by mild cognitive impairment or AD [18], [23]. Differences in IgM auto-antibodies have been suggested as a potential diagnostic marker, as the levels of specific IgM auto-antibodies were lower in AD patients compared to controls [18]. Interestingly also a catalytic degradation of Aβ has been described from IgM autoantibodies [20], [55]. However, regarding OMAB no such effect could be noted, data not shown.

All plasma samples from young healthy individuals screened by an indirect ELISA contained anti-Aβ IgM auto-antibodies with a strong preferential binding to oligomers as compared to monomers and dimers (Fig. 5B). The fact that these antibodies are ubiquitously present in normal plasma suggests that they have a possible role in the normal homeostasis of Aβ. The L11.3 antibody is derived from an Epstein-Barr virus immortalized cell line established from B lymphocytes of a healthy individual. Thus, L11.3 resembles an anti-Aβ antibody naturally present in the sera of healthy humans. Our finding that OMAB, L11.3, and, IgM auto-antibodies from eight plasma samples showed a very high selectivity towards Aβ oligomers suggests that human IgM auto-antibodies may confer a protective effect *in vivo*, both normally and when used for AD passive immune therapy.

The current work proposes that the IgM molecule provides an efficient architecture for the selective trapping of Aβ oligomers. The mechanism is mediated by the combined effect of avidity and a high on and off-rate at each single binding site. Taken together this generates an efficient, selective and also general mode of screening for oligomers. Once the oligomeric form is bound the interaction, as a result of avidity, becomes very strong. Through this mechanism antibody binding to monomers and precursor proteins like APP can be minimized. We also show that oligomer specificity appears to be a general feature of common human anti-Aβ IgM autoantibodies. A more extensive analysis comparing the oligomer specificity of natural occurring IgM anti-Aβ antibodies between groups of healthy donors and AD patients is currently ongoing.
